# Influenza A Virus Assembly Intermediates Fuse in the Cytoplasm

**DOI:** 10.1371/journal.ppat.1003971

**Published:** 2014-03-06

**Authors:** Seema S. Lakdawala, Yicong Wu, Peter Wawrzusin, Juraj Kabat, Andrew J. Broadbent, Elaine W. Lamirande, Ervin Fodor, Nihal Altan-Bonnet, Hari Shroff, Kanta Subbarao

**Affiliations:** 1 Laboratory of Infectious Diseases, National Institute of Allergy and Infectious Diseases, National Institutes of Health, Bethesda, Maryland, United States of America; 2 Section on High Resolution Optical Imaging, National Institute of Biomedical Imaging and Bioengineering, National Institutes of Health, Bethesda, Maryland, United States of America; 3 Research Technologies Branch, National Institute of Allergy and Infectious Diseases, National Institutes of Health, Bethesda, Maryland, United States of America; 4 Sir William Dunn School of Pathology, University of Oxford, Oxford, United Kingdom; 5 Laboratory of Host-Pathogen Dynamics, Cell Biology and Physiology Center, National Heart Lung and Blood Institute, National Institutes of Health, Bethesda, Maryland, United States of America; University of North Carolina at Chapel Hill, United States of America

## Abstract

Reassortment of influenza viral RNA (vRNA) segments in co-infected cells can lead to the emergence of viruses with pandemic potential. Replication of influenza vRNA occurs in the nucleus of infected cells, while progeny virions bud from the plasma membrane. However, the intracellular mechanics of vRNA assembly into progeny virions is not well understood. Here we used recent advances in microscopy to explore vRNA assembly and transport during a productive infection. We visualized four distinct vRNA segments within a single cell using fluorescent *in situ* hybridization (FISH) and observed that foci containing more than one vRNA segment were found at the external nuclear periphery, suggesting that vRNA segments are not exported to the cytoplasm individually. Although many cytoplasmic foci contain multiple vRNA segments, not all vRNA species are present in every focus, indicating that assembly of all eight vRNA segments does not occur prior to export from the nucleus. To extend the observations made in fixed cells, we used a virus that encodes GFP fused to the viral polymerase acidic (PA) protein (WSN PA-GFP) to explore the dynamics of vRNA assembly in live cells during a productive infection. Since WSN PA-GFP colocalizes with viral nucleoprotein and influenza vRNA segments, we used it as a surrogate for visualizing vRNA transport in 3D and at high speed by inverted selective-plane illumination microscopy. We observed cytoplasmic PA-GFP foci colocalizing and traveling together en route to the plasma membrane. Our data strongly support a model in which vRNA segments are exported from the nucleus as complexes that assemble en route to the plasma membrane through dynamic colocalization events in the cytoplasm.

## Author Summary

Influenza A viruses, containing eight single stranded RNA segments, cause seasonal epidemics and occasional pandemics. Reassortment of the influenza viral genome in co-infected cells confers an evolutionary advantage for the virus, and can result in viruses with pandemic potential like the 2009 pandemic H1N1 and 2013 H7N9 virus. Replication of the viral genome occurs in the nucleus of the host cell and the progeny viral RNA (vRNA) segments must be transported to the plasma membrane for budding. The dynamics of vRNA assembly into progeny virions remains unknown. We used novel techniques to visualize the 3D-localization of four distinct vRNA segments in an infected cell and a fluorescent virus to visualize vRNA transport during a productive infection to determine where, when and how assembly occurs. Our data suggest that vRNA segments are exported from the nucleus as subcomplexes that undergo additional assembly en route to the plasma membrane through dynamic fusion events of vRNA-containing cytoplasmic foci. These observations have broad implications for understanding the intracellular requirements behind reassortment of influenza viruses and may lead to the development of new antiviral targets.

## Introduction

Influenza viruses cause severe annual morbidity and mortality [1]. The genome of influenza A viruses is composed of 8 negative-sense single stranded RNA gene segments (PB2, PB1, PA, HA, NP, NA, M and NS) that encode 10 major proteins and several auxiliary peptides. Production of infectious progeny virions requires incorporation of all 8 vRNA segments and occurs at the apical membrane of infected cells [2]. The segmented nature of the viral genome allows for the generation of novel reassortant viruses containing genes from distinct parental viruses in co-infected cells. The 2009 pandemic H1N1 virus arose from a reassortment event between two swine origin viruses [3]. Additionally, the 2013 H7N9 virus responsible for the outbreak in Mainland China and Taiwan is a reassortant virus deriving gene segments from avian H9N2 and H7N9 viruses [4]. Reassortment provides an evolutionary advantage for influenza viruses and thus reassortant viruses pose a major public health risk. Understanding how vRNA segments are assembled and packaged into progeny virions is key for unlocking how reassortant viruses are generated, yet little is known about this process.

Replication of the influenza virus genome occurs in the nucleus by a virally encoded heterotrimeric RNA-dependent RNA polymerase composed of PB1, PB2, and PA proteins [2]. Each newly synthesized vRNA segment is coated with the viral nucleoprotein (NP) and the heterotrimeric polymerase complex associates with each vRNA segment via the 5′ and 3′ ends. This resultant vRNP complex is exported from the nucleus and travels to the plasma membrane for packaging into a budding virion [2], [5]. Recent studies have suggested a role for Rab11a-containing recycling endosomes in the transport of vRNA segments to the plasma membrane for packaging [6]–[9]. However, the dynamics of vRNA transport, including whether vRNA segments are transported individually or as a complex, remain largely unclear.

Studies using electron tomography or fluorescent *in situ* hybridization (FISH) on purified influenza viral particles suggest that the majority of progeny virions incorporate a single copy of all 8 vRNA segments [10]–[13]. Selective packaging of all 8 segments into progeny virions is thought to occur via RNA-RNA interactions between the vRNA segments identified through *in vitro* studies, which suggests the formation of an interaction network between vRNA segments [5], [11], [13]–[16]. However, the organization and mechanics of vRNA assembly are poorly understood, due mostly to the lack of adequate tools for observing the transport and spatial arrangement of vRNA segments *in situ*.

Visualization of intracellular vRNA segments has been limited to two-color FISH [12], [17], hindering assessment of the localization and interactions between more than two vRNA segments within a single cell. Additionally, tracking vRNA movement during a productive infection requires a virus that expresses a fluorescent tag fused to a viral protein involved in vRNA trafficking, which is packaged into progeny virions and is maintained in subsequent generations. Although some fluorescent influenza viruses have been generated, they either contain GFP fused with a viral protein not involved in vRNA transport or as a separate polypeptide used as a marker for infection that fails to highlight any specific process of the viral lifecycle [18]–[20]. An influenza virus with PB2 tagged with split-GFP was recently used to assess the movement of vRNA [21]. However, while split-GFP is useful in studying protein-protein interactions, it is a poor choice for visualizing protein dynamics because 1) visualization is limited to cells expressing the remainder of the GFP molecule; 2) visualization is not possible prior to formation of the complete GFP; 3) split-GFP fragments are unstable compared to intact GFP; and 4) split-GFP is prone to aggregation [22]–[24].

To explore the mechanics of vRNA assembly we developed two novel imaging tools: a system to visualize four different vRNA segments within an infected cell and a fluorescent influenza virus to track vRNA movement in live cells during a productive infection. Using the four-color FISH assay we were able to visualize all 8 vRNA segments and quantify the number of cytoplasmic foci and found that the majority of the foci at the external nuclear periphery contain more than one vRNA segment. To assess the dynamics of vRNA assembly in the cytoplasm we generated an influenza virus, A/WSN/1933 (H1N1) (WSN) expressing full-length GFP fused to the viral polymerase PA protein (WSN PA-GFP) and observed that PA-GFP foci colocalize and travel together in the cytoplasm by an inverted selective-plane illumination microscope. Our data provide evidence that vRNA segments form subcomplexes throughout the cytoplasm during infection, that these subcomplexes contain multiple distinct vRNA segments, and that vRNA-containing cytoplasmic foci can fuse together during transport.

## Results

### Visualization of multiple vRNA segments within a single infected cell

**Figure 1:**
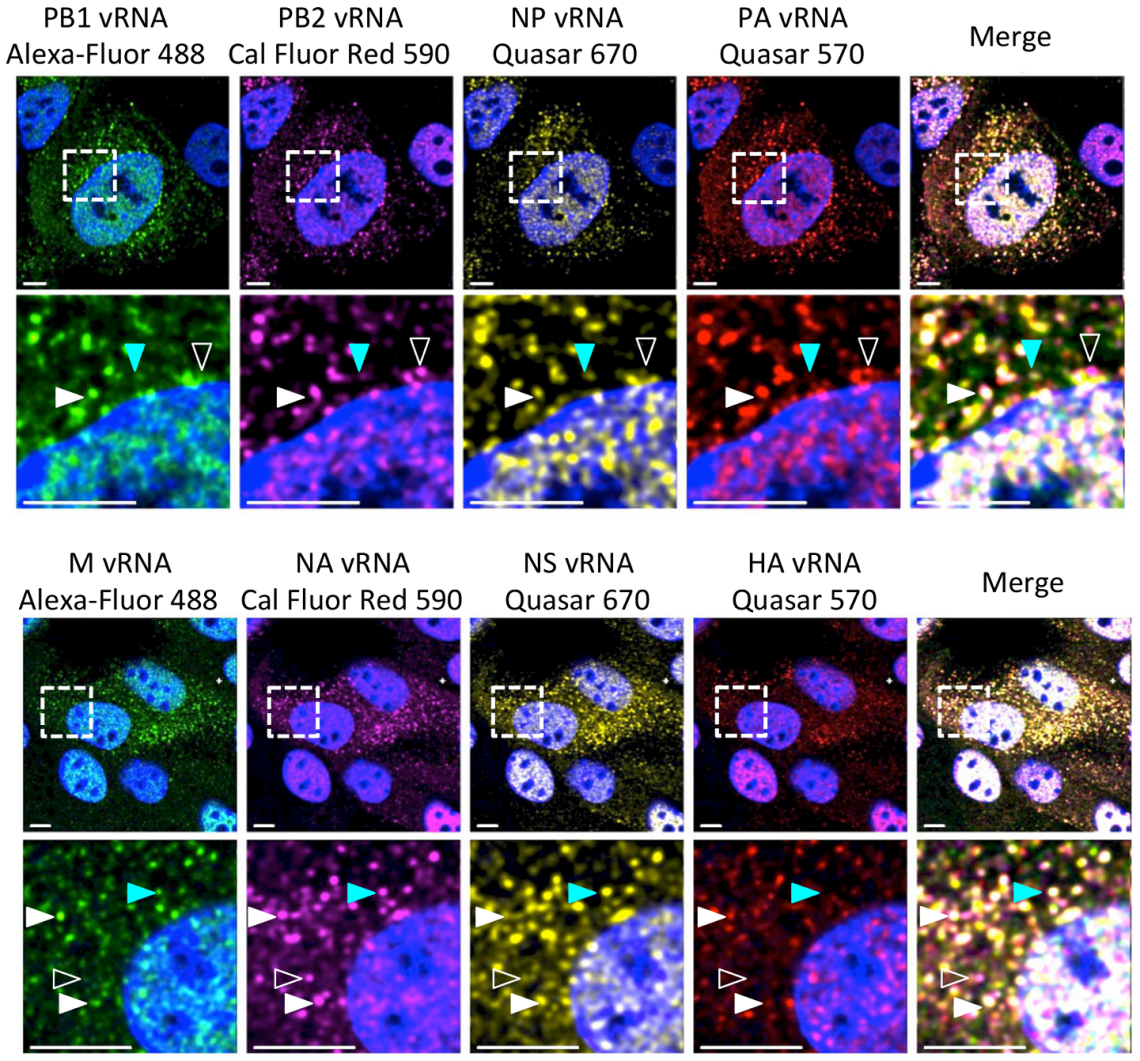
Visualization of four distinct influenza viral gene segments within a single cell. MDCK cells were infected with wild type (WT) WSN at a multiplicity of infection (MOI) of 3 for 8 hours post infection (hpi) and then imaged with FISH probes directed against four distinct vRNA species. The first row displays a cell probed for influenza vRNA segments PB2, PB1, PA, and NP. Row 3 contains images of a cell probed for the other 4 influenza vRNA segments: HA, NA, M and NS. The second and fourth row of images show an enlargement of the area defined by the dashed boxes in the first and third rows. Solid white arrowheads identify a cytoplasmic focus with all four distinct vRNA segments, turquoise arrowheads indicate a focus with only three vRNA species, and open arrowheads show a focus with only two vRNA species. All scale bars are 10 µm. DAPI marks the cellular nucleus.

In order to explore where vRNA assembly occurs during a viral infection we established a system to simultaneously visualize four different vRNA segments within an infected cell (Figure 1). We visualized four distinct fluorophores conjugated to vRNA probes with a tunable white light laser confocal microscope, using a sequential scanning program to ensure minimal cross talk between channels (Figure S1). We designed probes against all 8 vRNA segments of A/WSN/1933 (H1N1) virus to enable visualization of all 8 segments in relation to each other in multiple four-color FISH experiments. Figure 1 depicts two representative cells displaying all 8 vRNA segments; one cell probed for vRNA segments PB2, PB1, PA, and NP and the other probed for HA, NA, M and NS vRNA segments. We confirmed the specificity of each FISH probe set by testing each of the probes on cells expressing a single vRNA transcript from a PolI expression plasmid (Figure S2). To our knowledge, our study is the first to visualize four distinct vRNA species within a single cell.

**Figure 2:**
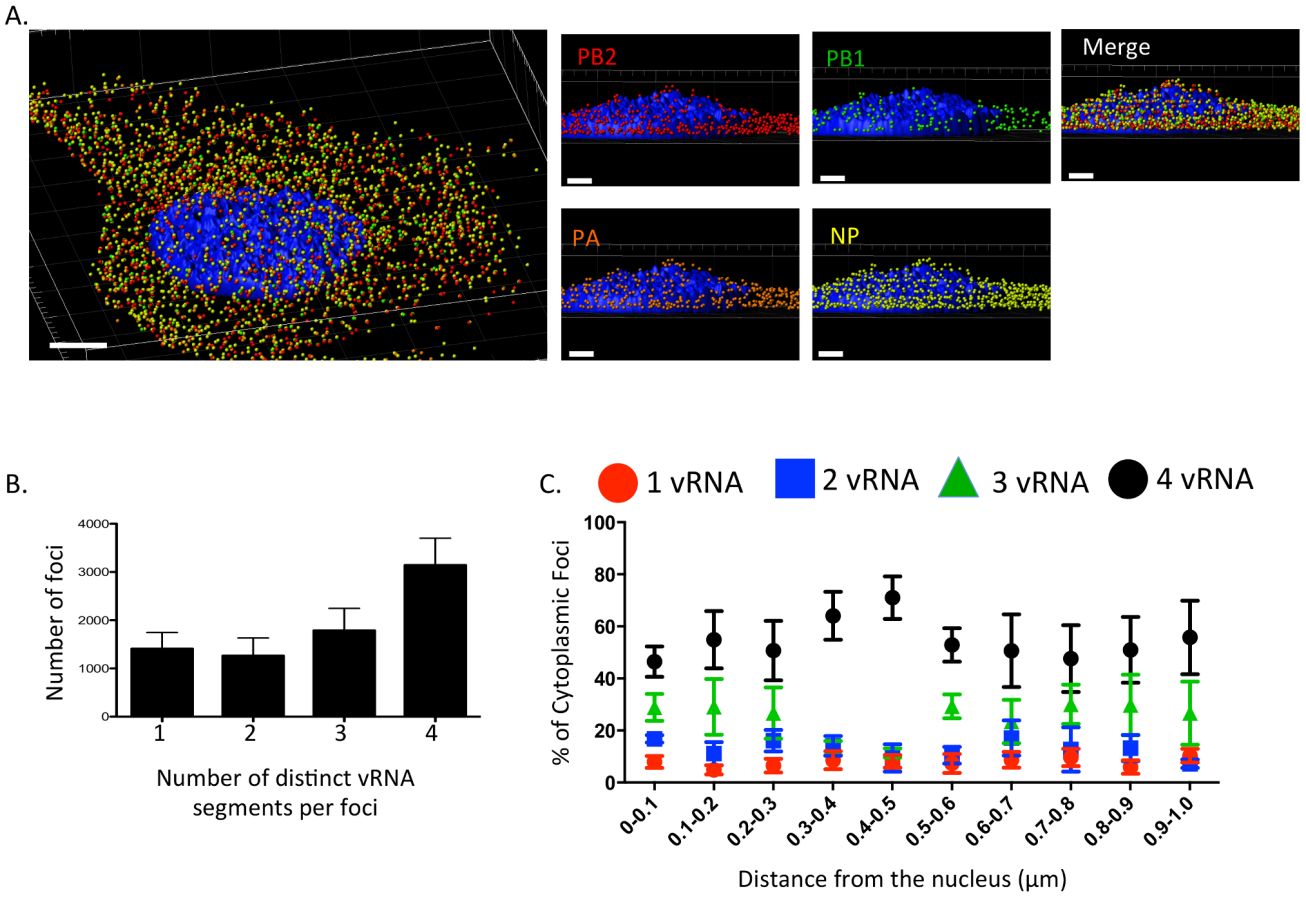
Viral RNA composition and spatial location of cytoplasmic foci. 3D rendering of an MDCK cell infected with WT WSN (MOI = 3) for 8 hpi were stained with probe B from Table S1: WSN PB2 Quasar 570 (red spots), PB1 Alexa-Fluor 488 (green spots), PA Cal Fluor Red 590 (orange spots), and NP Quasar 670 (yellow spots) (A). The DAPI-stained nucleus is labeled in blue. An enlarged region of the 3D image was tilted 90° in the z-direction to provide an axial-view of the cytoplasmic foci and nucleus. Scale bars are 4 µm in the whole cell image and 2 µm in the rotated images. The total number of foci containing 1, 2, 3, or 4 vRNA segments was quantified within a given cell (B). Each bar represents the average from 3 independently analyzed cells with standard error indicated. The distance from the nucleus for each focus from four independent cells was calculated (C). The proportion of foci containing 4,3,2,or 1 distinct vRNA segment with a given range from the nucleus is represented graphically as a scatter plot. Each spot is an average from 4 independent cells and the standard error is indicated.

Staining of vRNA in WT WSN infected Madin-Darby canine kidney (MDCK) cells using four-color FISH displayed diffuse nuclear staining and distinct cytoplasmic foci. Closer inspection of the cytoplasmic foci in the enlarged areas of Figure 1 revealed foci containing combinations of 4, 3, 2, or a single vRNA segment (various arrowheads). Staining of vRNA segments in the nucleus indicated a high degree of colocalization between the vRNA foci (white pixels in the merged nucleus image); however, the high density of the vRNA segments and their diffuse appearance prevented accurate assessment of potential colocalization between vRNA segments in the nucleus. Therefore, we focused on the fate of vRNA segments after nuclear export. To explore the composition of each cytoplasmic focus we used the imaging software Imaris to mask the nucleus, thus excluding the vRNA nuclear staining from our analysis. Figure 2A shows a 3D rendering of all the cytoplasmic foci in a MDCK cell infected with WT WSN and probed for PB2 (red), PB1 (green), PA (orange), and NP (yellow), which encode proteins that form the viral RNP complex. This image was rotated to provide an axial view of the cell and to highlight the cytoplasmic foci in relationship with the nucleus. To quantify the total number of foci containing a single labeled vRNA segment, 2 distinct vRNA segments, 3 different segments, or all 4 vRNA segments, we determined the signal intensity for each fluorophore in each cytoplasmic focus from three independent cells. We found that a significantly larger number of cytoplasmic foci contained all 4 labeled vRNA segments (Figure 2B). This observation was consistent for each set of four vRNA segments that we tested (Figure S3).

To determine whether the proportion of cytoplasmic foci containing 4, 3, 2, or 1 vRNA segments changed spatially in relationship to the nucleus, we determined the 3D distance from the nucleus for each focus using the DAPI signal to define the nuclear boundary. We found that the majority of the foci near or at the nucleus (within 0.1 µm) contained more than one vRNA segment (Figure 2C), suggesting that vRNA segments are not exported from the nucleus as individual segments. In general, independent of distance from the nucleus the proportion of foci containing 3 or 4 vRNA segments was much higher than those containing 1 or 2 vRNA segments (Figure 2C). Interestingly, we observed an increase in the proportion of foci containing four vRNA as the distance from the nucleus increased to 0.5 µm (Figure 2C). Based on the axial view of the cell in Figure 2A, the top of nucleus is very close to the apical cellular membrane, as defined by FISH staining. There may be a bias at 0.5 µm from the nucleus for foci that are at the plasma membrane being incorporated into progeny virions preparing to bud. The predominance of foci containing all four labeled vRNA segments close to the apical surface of the cell is consistent with the notion that vRNA assembly is selective and that there is a mechanism to ensure packaging of all 8 vRNA segments. However, since the distance from the nucleus to the cellular membrane is not uniform in all directions, we observed many foci located at distances greater than 0.5 µm from the nucleus that did not contain all 4 of the labeled vRNA segments (Figure 2C).

### Colocalization of vRNA segments

**Figure 3:**
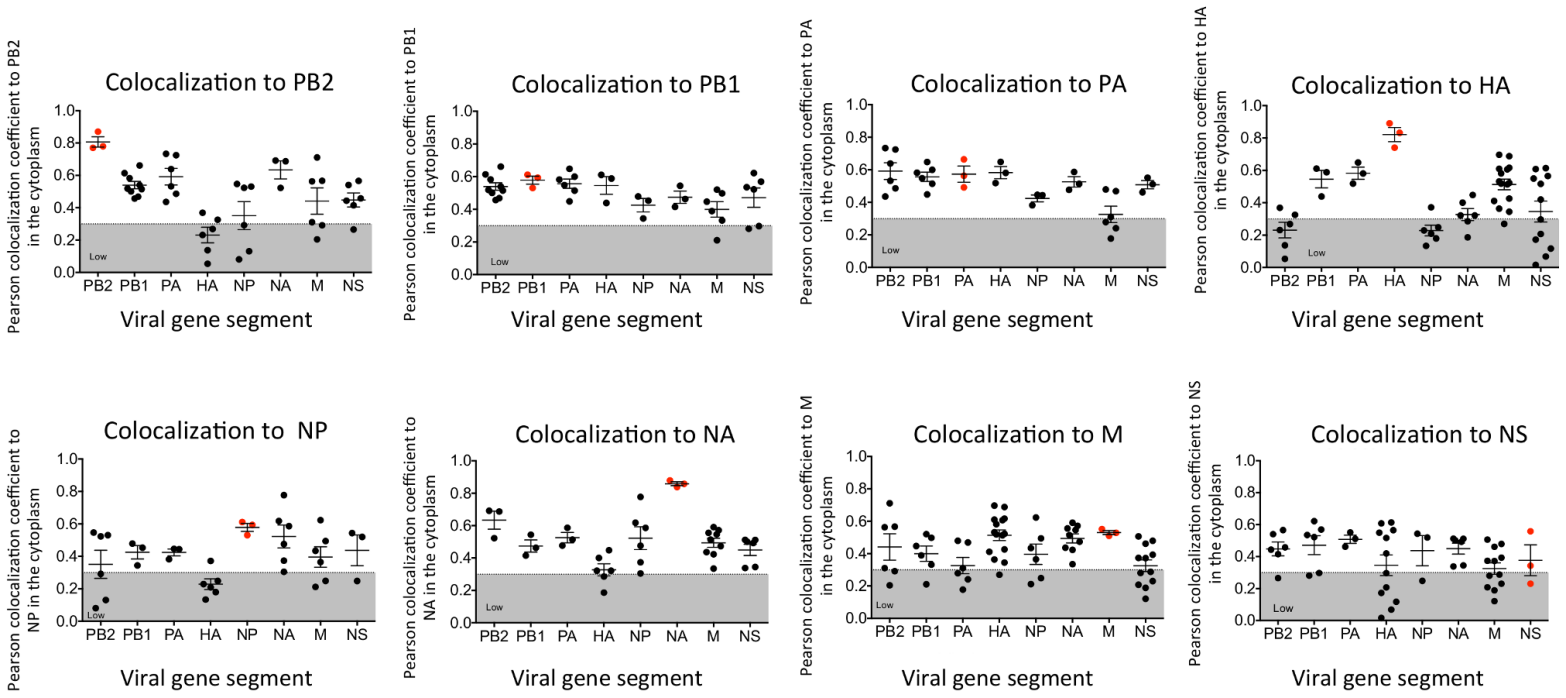
Colocalization coefficients between influenza vRNA segments. Graphical representation of the Pearson correlation coefficient values between each vRNA segment and all other vRNA segments from exclusively cytoplasmic staining. Dual staining of infected cells with FISH probes against the same target served as a positive control and is indicated in red. Each spot represents a single analyzed infected cell. Gray shading highlights the region of low colocalization based on the Pearson correlation coefficient.

In vitro studies have shown that vRNA segments associate with each other [13], [15], [16], suggesting that selective packaging of vRNA segments is mediated via vRNA-vRNA interactions. A proposed mechanism for assembly includes the formation of a single linear interaction network between vRNA segments for packaging [5]. We multiplexed FISH probes (Table S1) to compare each vRNA segment to each other in an attempt to identify a linear interaction network between vRNA segments, and calculated the Pearson correlation coefficient between each vRNA segment in the cytoplasm (Figure 3). The Pearson correlation coefficient has been used extensively for estimating the colocalization between two channels [25] as it suggests the degree of colocalization: values of 0.5–1 are considered high, 0.3–0.5 moderate, and 0–0.3 low. As a positive control, we probed the same segment with two FISH probes conjugated to different fluorophores. The Pearson coefficient for the positive controls was in the high range, with the exception of the NS gene segment (Figure 3). In general we observed colocalization coefficient values between vRNA segments in the high to moderate range, making it difficult to ascribe a linear order of assembly between vRNA segments. Interestingly, we observed a low correlation of ∼0.2 between PB2 and HA vRNA segments (Figure 3). This observation is of particular interest since these two gene segments encode the most well characterized virulence motifs of influenza viruses [26]–[29].

### Generation of WSN PA-GFP virus

**Figure 4:**
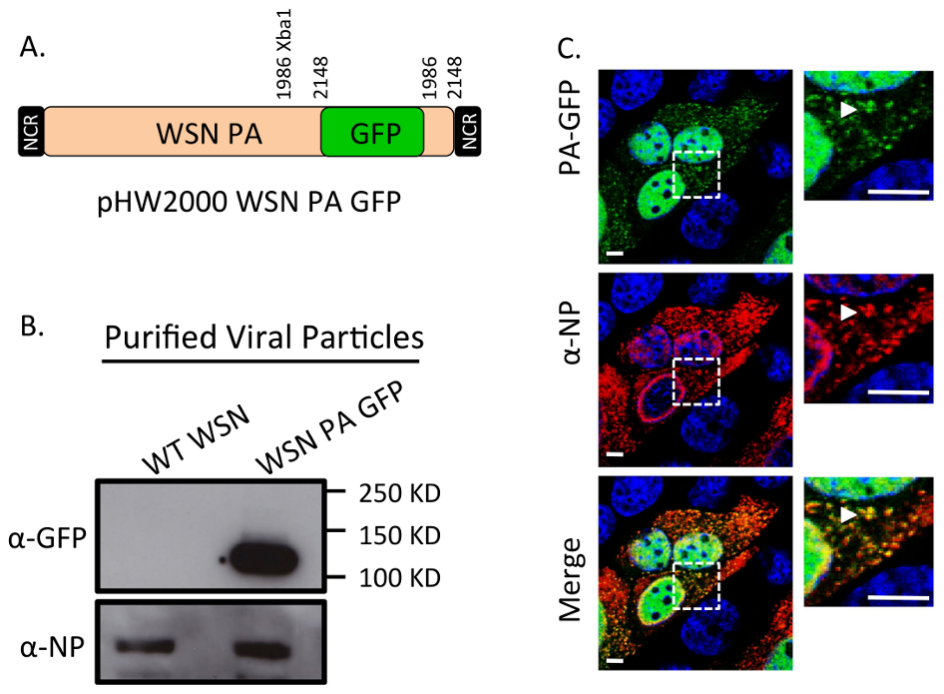
Generation of WSN PA-GFP influenza virus. Schematic of the reverse genetics pHW2000 plasmid created by inserting the coding region of full length GFP into the C-terminal domain of the WSN PA open reading frame (A). This insertion included a duplication of 162 nucleotides of WSN PA prior to the 5′ non-coding region (NCR) depicted in the black rectangle. Western blot of purified recombinant WT WSN or WSN PA-GFP virus grown in embryonated eggs (B). The GFP signal corresponds to the appropriate size of a PA-GFP fusion protein. Viral NP protein was used as a control to ensure loading of equivalent amounts of virus. Immunofluorescence of MDCK cells infected with WSN PA-GFP (MOI = 3) for 16 hpi with anti-influenza NP antibody (C). Images to the right are enlarged regions identified by the dashed square. White arrows show areas of co-localization. All scale bars are 5 µm.

The four-color FISH studies suggest that vRNA segments are exported from the nucleus as subcomplexes that assemble en route to the plasma membrane. In order to investigate this further, we sought to observe cytoplasmic vRNA trafficking in live cells. Since it is technically challenging to label vRNA segments for live cell imaging, we developed a fluorescent virus that could be used to study viral replication during a productive infection. We generated a WSN virus encoding a full length GFP fused to the viral PA protein, a component of the heterotrimeric viral polymerase complex with endonuclease activity that is associated with the 3′ and 5′ ends of vRNA segments [2], [30]–[32]. Creation of the WSN PA-GFP virus built upon two previously published observations. First, the WSN PA protein, fused to GFP at its C-terminus, functions like wild-type (WT) PA protein in *in vitro* replication experiments [33]. Second, rescue of viruses with a vRNA segment containing a large fragment of foreign RNA requires duplication of approximately 150 base pairs (bp) of the coding region prior to the 3′ non-coding region [34]. Thus, we generated a WSN PA-GFP virus with a portion of the C-terminal coding region duplicated after the GFP stop codon and prior to the non-coding region of the PA gene segment (Figure 4A).

**Figure 5:**
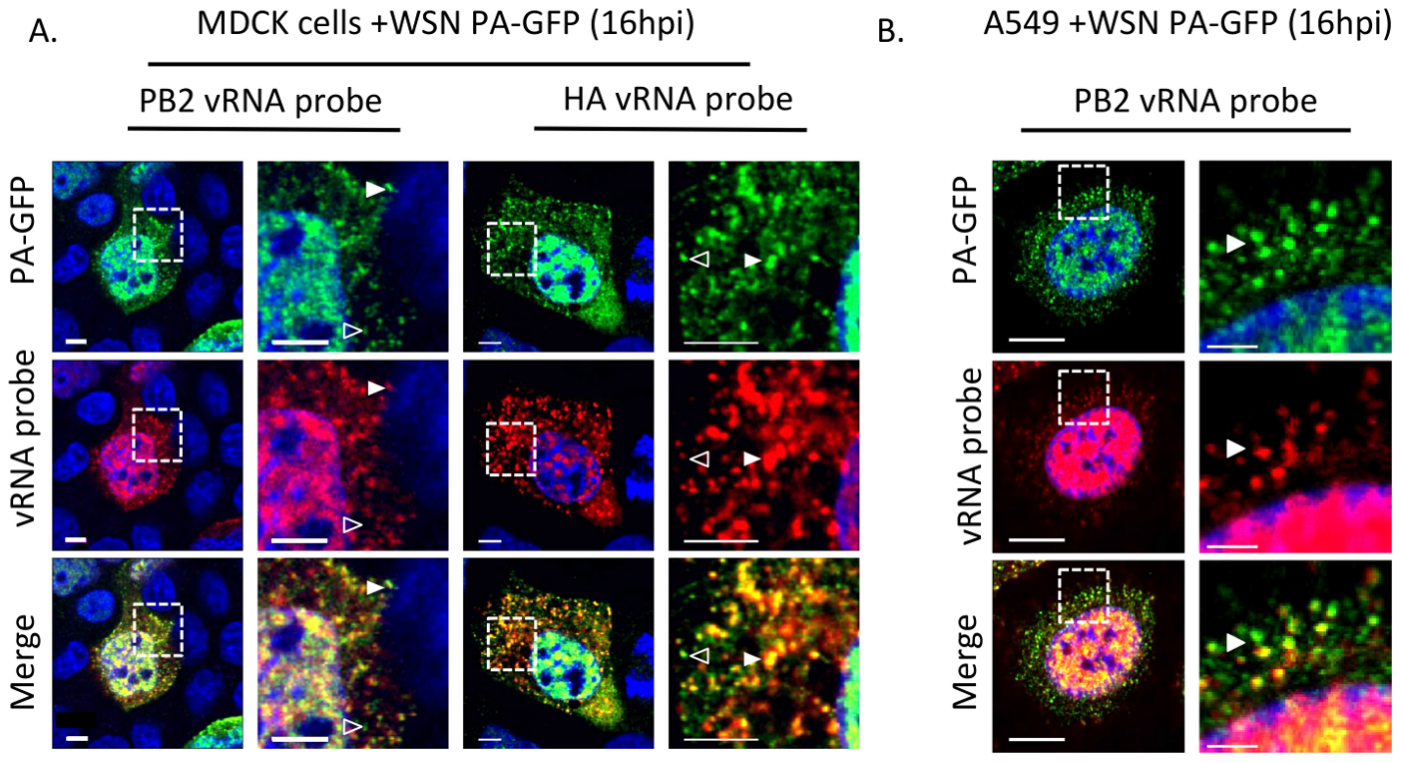
Colocalization of WSN PA-GFP and influenza vRNA segments. Fluorescent *in situ* hybridization (FISH) for viral RNA segments 1 (PB2) and 4 (HA) on MDCK (A) or A549 cells (B) cells 16 hpi with WSN PA-GFP (MOI = 0.5). Images to the right are enlarged regions identified by the dashed box. White arrowheads show colocalization of PA-GFP and vRNA, and open arrowheads indicate GFP foci not colocalized with viral RNA signal. DAPI marks the cellular nucleus. Scale bars are 5 µm in all panels of part A. In part B scale bars are 10 µm in the whole cell images and 2 µm in length in the enlarged region.

Western blot analysis of purified WT WSN and WSN PA-GFP virions demonstrate that GFP is present in mature virions and runs slightly above the 100 kDa marker, which is appropriate for a PA-GFP fusion since PA is 82 kDa and GFP is about 35 kDa (Figure 4B). Presence of the viral NP protein was used to ensure equal loading of both viruses. The localization pattern of WSN PA-GFP in MDCK cells 16 hours post-infection was diffusely nuclear with discrete cytoplasmic foci that colocalized with the viral NP protein (Figure 4C). We confirmed that PA-GFP colocalized with vRNA segments by using FISH against vRNA segments PB2 and HA in both MDCK (Figure 5A) and human lung adenocarcinoma epithelial (A549) cells (Figure 5B) 16 hours post-infection. Additionally, treatment of infected cells with leptomycin B (LMB) 4 hours post-infection restricted the localization of PA-GFP, NP, and vRNA segments to the nucleus 24 hours post-infection (Figure S4). Nuclear retention of these vRNP components in the presence of LMB demonstrates that nuclear export depends upon exportin 1 (Crm1), as previously suggested [35]–[37]. Taken together, our data demonstrate that PA-GFP can serve as a surrogate for vRNA gene segments in cells infected with the WSN PA-GFP virus.

### Characterization of PA-GFP movements during productive viral infection

Previous studies exploring the dynamics of vRNA transport using transient transfection systems or the PB2 split-GFP virus have described both fast (∼1 µm/s) and slow (∼0.12 µm/s) movements [7], [21]. In order to capture the 3D dynamics spanning a large temporal range, while minimizing photobleaching and photodamage, we used the recently developed inverted selective-plane illumination microscopy (iSPIM) [38] to image the movement of PA-GFP foci every 2 seconds for 30 minutes in MDCK and A549 cells starting 16 hours post-infection (Movies S1 and S2). Multiple WSN PA-GFP trajectories were analyzed from each cell. In order to define the movement of the foci we determined the mean squared displacement (MSD) of each PA-GFP focus during a trajectory [39]. We found that many foci displayed diffusive movement, based on the characteristic linear dependence of their MSDs on time, while a quarter of the foci appeared to undergo active transport, as their MSDs displayed nonlinear time dependence (Figure S5). PA-GFP trajectories with active transport had average speeds of 0.23+/−0.06 µm/s and 0.27+/−0.11 µm/s in MDCK and A549 cells respectively. These speeds are similar to the maximum speeds of microtubule-mediated endosomal transport in a HeLa-derived cell line (0.25–1.5 µm/s) [40].

Closer inspection of PA-GFP tracks revealed that many foci colocalized during their trajectories. Strikingly, we observed that foci displayed distinct fates after colocalization. Some foci appeared to come together and persist as a single diffraction-limited spot for the remainder of the trajectory (herein referred to as ‘fusion’) while other foci transiently colocalized, then separated and continued on as distinct foci. We quantified the number of fusion and transient colocalization events in WSN PA-GFP infected MDCK and A549 cells (Tables S2 and S3). Most trajectories displayed transient colocalization events (69% and 88% in MDCK and A549 cells, respectively), whereas fusion events were relatively rare (9% and 16% for MDCK and A549 cells, respectively).

**Figure 6:**
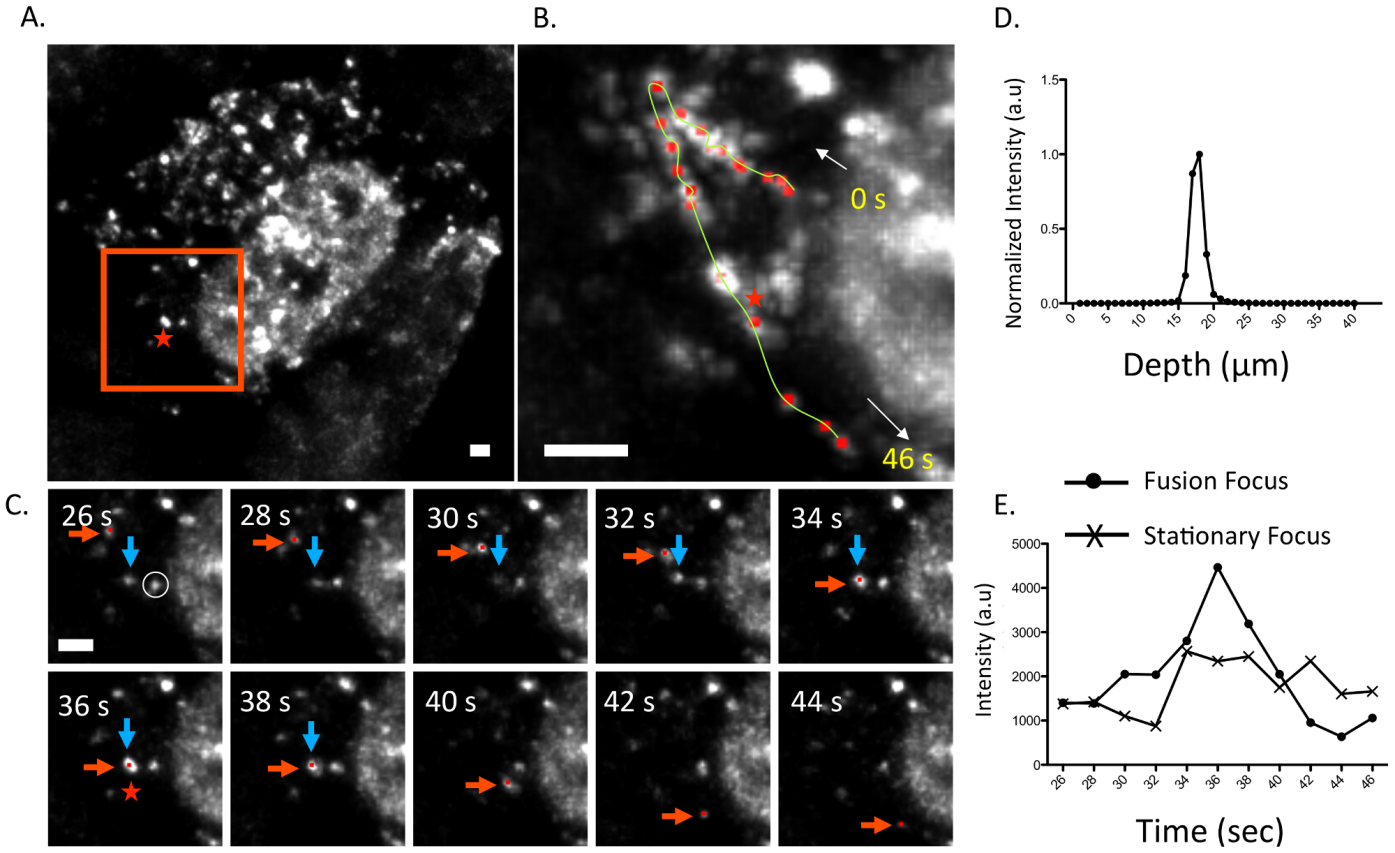
Fusion event of an WSN PA-GFP focus in the cytoplasm of MDCK cells. Maximum intensity projection from inverted selective-plane illumination microscopy (iSPIM) movie of MDCK cells infected with WSN PA-GFP (MOI = 5) at 16 hpi, 36 seconds (s) after onset of tracking (A). The red-boxed region is highlighted in (B and C), and the red star indicates the region where colocalization of two GFP foci (marked in red and blue arrows) occurred. A single WSN PA-GFP focus trajectory; each red dot is a spot tracked in time, the yellow line is the overall trace, and white arrows indicate the direction of the PA-GFP movement (B). The total track duration is 46 s. Part C contains still frames of the red-boxed region in part A from 26–44 s, and illustrates colocalization and subsequent fusion of two foci. The red arrow indicates the PA-GFP focus that is being tracked and the blue arrow indicates the PA-GFP focus with which it fuses. All scale bars are 2 µm. An intensity versus depth plot of the colocalized foci, indicated by the red star in part A, demonstrates a single peak indicating that the fusion occurs in a single diffraction-limited spot (D). An intensity vs. time plot of the fused focus compared to an adjacent stationary focus, identified by the white circle in part C, during the same time frame (E). The asterisk marks the time corresponding to the colocalization event.

Figure 6 depicts an example of a fusion event involving two cytoplasmic PA-GFP foci within an infected MDCK cell (Figure 6A). A single focus is tracked from near the nuclear edge into the cytoplasm and towards the cell periphery (Figure 6B). After 36 s, the PA-GFP focus (Figure 6C, red arrow) colocalized and fused with another focus (blue arrow). We confirmed that these two foci persisted as a single focus after colocalization by observing 1) a single peak in a fluorescence intensity vs. depth plot at the site of fusion and 2) an increase in fluorescence intensity of the fused focus at the time of fusion compared to a stationary, unfused adjacent focus (Figure 6D and E). While previous studies reported similar average speeds of vRNA trafficking [21] they did not observe dynamic fusion and colocalization events in the cytoplasm. The WSN PA-GFP virus enabled us to extend these previous studies and describe a novel aspect of vRNA transport. The fusion of multiple PA-GFP foci in the cytoplasm may reflect the coalescence of polymerase complexes attached to different vRNA segments, suggesting that the subcomplexes we identified using four-color FISH dynamically associate with each other in the cytoplasm en route to the plasma membrane.

## Discussion

**Figure 7:**
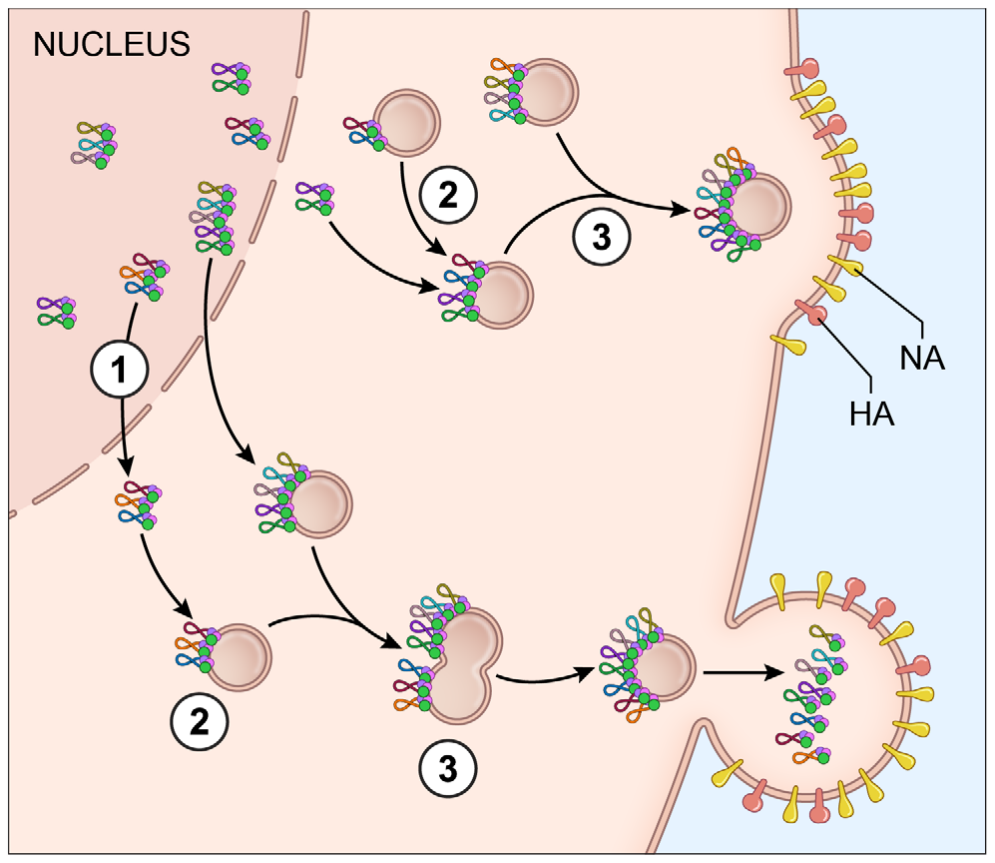
Proposed model for vRNA assembly. The data presented in this paper supports a model of vRNA assembly whereby 1) vRNA segments export from the nucleus as complexes containing multiple vRNA segments, 2) cytoplasmic foci containing vRNA segments transport through the cytoplasm and 3) these cytoplasmic foci fuse together in the cytoplasm prior to budding. Each vRNA segment is shown as a loop where the ends are associated with a heterotrimeric polymerase complex identified as 3 circles. The PA protein of the polymerase complex is shown in green since this was the protein used as a surrogate for vRNA transport. The cellular Rab11a-containing endosomes that colocalize with vRNA-containing cytoplasmic foci are indicated.

Reassortment of influenza virus gene segments can lead to the emergence of novel influenza viruses with pandemic potential. The data from this study provide insight into vRNA assembly, which is important for understanding the intracellular mechanism of vRNA reassortment. First, our data suggest that vRNA segments are not exported as individual segments since the majority of foci at the external nuclear periphery contain more than one vRNA segment (Figure 2C). Second, we observed many cytoplasmic foci with fewer than 4 vRNA segments (Figure 1, 2A, 2C and Figure S3), implying that all 8 vRNA segments are not exported from the nucleus together. Third, individual vRNA segments do not reach the plasma membrane separately since many foci contain more than a single vRNA segment and foci can fuse together in the cytoplasm (Figure 1, 2B, and 6C). Therefore, we believe that vRNA assembly is an active process that includes the formation of flexible subcomplexes that export from the nucleus and then undergo further assembly en route to the plasma membrane via dynamic colocalization events (Figure 7).

Electron tomography has shown that vRNA segments within progeny virions have a conserved ‘7+1’ structure, where 7 segments surrounding a center segment [11], [13]. Selective assembly of vRNA segments into this type of conformation would seem to require either a linear ordering of vRNA-vRNA associations or a the existence of a master segment in the center that can interact with all other segments [5]. *In vitro* studies exploring the RNA-RNA interactions between all 8 segments have suggested that indeed a linear interaction network between vRNA segments is used for vRNA assembly [13], [16], but these interaction networks are not conserved between different subtypes of influenza A viruses [15]. In this study, we attempted to identify preferences between vRNA segments using the four-color FISH assay and found that most vRNA segments colocalize with each other with a high confidence value; we did not observe a distinct linear preference between vRNA segments (Figure 3). We believe that the high specificity of each FISH probe set (Figure S2) limits the likelihood of false positive results in our colocalization studies. In addition, our data are based on the colocalization profiles of vRNA segments throughout the entire cytoplasm, including the plasma membrane where vRNA segments are localized in high concentration prior to budding. Therefore, our data neither support nor refute the hypothesis that vRNA segments associate in an ordered fashion. Further analysis that carefully examines the colocalization profiles of vRNA segments and the vRNA composition of the cytoplasmic foci at different distances from the nucleus would provide spatially defined colocalization coefficients and interaction networks, which may help to clarify the nature of the selective arrangement between vRNA segments.

Interestingly, we observed a low correlation coefficient between the HA and PB2 vRNA segments, which encode known viral host range determinants [29]. HA mediates attachment and entry into host cells by binding to terminally attached sialic acid moieties on cell-surface glycoproteins; avian influenza viruses predominantly associate with α2,3-linked sialic acids while human viruses bind α2,6-linked sialic acids [41], [42]. Multiple residues of the PB2 protein, a component of the viral heterotrimeric polymerase complex, restrict replication of the virus in mammalian cells; for example the residue at position 627 typically encodes glutamic acid in avian influenza viruses and lysine in most human influenza viruses [26], [43], [44]. Interestingly, the recent studies generating airborne transmissible avian H5N1 viruses described viruses where the HA protein was modified to associate with α2,6 sialic acid moieties and either contained a human PB2 vRNA segment or the PB2 627K residue [45]–[47], demonstrating the importance of these two gene segments in emerging avian influenza pandemic viruses. Based on our data, a low correlation coefficient between the HA and PB2 vRNA segments may indicate that these segments are less likely to reassort together than two segments with a high correlation coefficient like PB1 and HA vRNA, which reassorted together in the 1957 H2N2 pandemic virus [48].

The observations we present here extend and refine previous assumptions regarding vRNA assembly. A recent study [12] proposed a scheme for vRNA assembly based on visualization of only 2 vRNA segments (PB2 and NA), in which they suggest that vRNA segments are exported from the nucleus individually. By visualizing four vRNA segments simultaneously we demonstrate that most cytoplasmic foci contain more than one vRNA segment. While our data support the overall conclusion proposed by Chou et al that vRNA assembly occurs en route to the PM, we not only provide much stronger support for this model but we also extend the observations made in fixed cells by visualizing vRNA transport in live cells during a productive infection. We demonstrate that cytoplasmic foci dynamically colocalize with each other, potentially mediating further assembly of vRNA subcomplexes. In addition, previous studies have shown that influenza vRNA segments colocalize with recycling endosomes containing the small GTPase Rab11a, and that these endosomes facilitate vRNA trafficking in the cytoplasm [7], [9], [36]. We confirmed colocalization of Rab11a and influenza PB2 vRNA segment in A549 cells transfected with Rab11a-GFP (Figure S6). Since WSN PA-GFP foci colocalize with PB2 vRNA segment (Figure 5) it is likely that WSN PA-GFP and Rab11a also associate, suggesting that the dynamic colocalization events observed during viral infection might also involve Rab11a-containing recycling endosomes. Further studies characterizing the importance of the transient or sustained colocalization events in viral replication may reveal novel targets for antiviral therapeutics.

The results we present here provide insight into the dynamics of vRNA transport and assembly, but our methods have applications beyond vRNA trafficking. For example, the WSN PA-GFP virus can be used to observe the kinetics of spread of influenza virus in cells (Movie S3), because the fluorescence can be tracked for extended periods of time. Development of fluorescent HIV, coronavirus and measles viruses have greatly enhanced our understanding of the biology of these pathogens, from production of progeny virions to viral spread [49]–[51]. We believe the observations and tools described in this study will enable similar progress in understanding of influenza virus biology.

## Materials and Methods

### Cells and viruses

Madin-Darby Canine kidney (MDCK) epithelial cells and adenocarcinoma human alveolar basal epithelial cells (A549) obtained from ATCC were maintained in modified Eagle's medium (MEM) with 10% fetal calf serum (FCS) and L-glutamine or Dulbecco's MEM (DMEM) with 10% FCS respectively. 293T cells (from ATCC) were used to rescue recombinant viruses and were maintained in 10% FCS DMEM.

Recombinant A/WSN/1933 (H1N1) viruses were rescued using an eight-plasmid (pHW2000) reverse genetics system, a kind gift from Dr. Richard Webby of St. Jude Children's Research Hospital. WSN PA-GFP was constructed by engineering a silent Xba1 site in PA using site-directed mutagenesis (Stratagene Santa Clara, CA) into both pHW2000 WSN PA and pCDNA WSN PA-GFP [33] at position 1986 (WT PA 1984- TCA AGA – 1989; PA XBA1 1984- TCT AGA – 1989). The pCDNA WSN PA-GFP plasmid contained an Xba1 site just after the GFP stop codon, so this plasmid was cut with Xba1, and the C-terminal PA-GFP fusion was inserted into the pHW2000 WSN PA Xba1 digested vector. This strategy will result in a duplication of 165 base pairs of the WSN PA coding region after the GFP stop codon as depicted in Figure 4A. WT WSN and WSN PA-GFP viruses were rescued by transfection in 293T cells with TransIT transfection reagent (Mirus Bio Madison, WI) and then plaque purified on MDCK cells. GFP positive plaques were used to inoculate 10-day old embryonated chicken eggs. Infectivity titers for all virus stocks were calculated the 50% tissue culture infectious dose (TCID_50_) per mL as described in [52].

Western blot analysis for GFP (mouse anti-GFP antibody, Living Colors, Invitrogen 1∶1,000) or viral NP (mouse anti-Influenza A NP antibody, Millipore 1∶5,000) was performed on purified viruses amplified in embryonated egg allantoic fluid.

### Immunofluorescence

MDCK or A549 cells grown on circular coverslips (12Cir-1.5, Fisher Scientific Pittsburgh, PA) were infected with virus for the time and multiplicity of infection (MOI) specified in the figure legends. The cells were fixed with 4% paraformaldehyde (PFA) and probed with anti-Influenza A NP antibody (Millipore 1∶2,000) for one hour followed by Alexa Fluor-594 goat anti-mouse Fab' fragment (Invitrogen 1∶2,000) for visualization of viral NP protein. To determine the role of Crm1 on the export of WSN PA-GFP protein, cells were infected with WSN PA-GFP virus and treated 4 hours post infection (hpi) with leptomycin B (2.5 mg/mL) that was kept on for the remainder of the infection. Cells were fixed 16 hpi and stained as described above.

### Four color fluorescent *in situ* hybridization (FISH)

Probes for FISH were designed and purchased from BioSearch Technologies (Novato, CA) and were directed against each vRNA segment. Each probe is ∼20 base pairs and ∼30 probes cover the length of a given vRNA target. The sequence of each probe was manually compared against the entire WSN influenza genome to ensure specificity to the targeted vRNA segment; any probes with greater than 10 base pairs complementarity to another vRNA target or to a mRNA sequence were excluded prior to purchase. This rigorous evaluation of the FISH probe sequences allowed for high specificity of the FISH probes to the specific vRNA segment.

The fluorophores used in our four-color FISH experiments are: Quasar 570, CAL Fluor Red 590, Quasar 670, and Alexa-Fluor 488. FISH probes conjugated to the Alexa-Fluor 488 fluorophore were produced manually to amine-terminal oligos synthesized by BioSearch Technologies and conjugated using the Invitrogen Alexa-Fluor 488 Oligonucleotide Amine Labeling kit per the manufacturer's recommendations.

Infected cells were stained with FISH probes from BioSearch using a standard protocol previously described in [53]. Briefly, cells grown on circular coverslips were infected at an MOI = 3 and fixed 8 hpi with 4% PFA stored in ice cold 70% EtOH overnight. The next day the coverslips were rehydrated with the wash buffer (10% formamide and 2× SSC in DEPC-treated water). Cells were incubated overnight in 200 µl of hybridization buffer (10% dextran sulfate, 2 mM vanadyl-ribonucleoside complex, 0.02% RNA-free BSA, 1 mg/ml e.Coli tRNA, 2× SSC, and 10% formamide in DEPC treated water) with 2 µl of labeled probes (probe concentrations ranged from 2.5 µM–10 µM) and incubated overnight in a 28°C incubator. The following day, the hybridization buffer was removed and cells were incubated in wash buffer containing DAPI for 10 minutes, washed then mounted. All incubations were performed in the dark.

Spectral separation of the four fluorophores was achieved using a Leica SP5 white-light laser confocal microscope. A sequential scanning program with a line average of 4 between frames was established based on the manufacturer's excitation and emission spectra for each fluorophore. The following parameters were used: PMT1 (DAPI) UV laser (power = 4%) wavelength range 415–470 nm, PMT2 (Alexa-Fluor 488) 488 nm excitation wavelength (λ_ex_) (power = 10%) range 500–540 nm, PMT3 (Quasar 570) 545 nm λ_ex_ (12%) range 553–585 nm, PMT4 (Cal Fluor Red 590) 575 nm λ_ex_ (12%) range 590–635 nm, and PMT5 (Quasar 670) 647 nm λ_ex_ (12%) range 675–750 nm. Spectral separation was confirmed in each experiment with single color controls (infected cells stained with only a single vRNA FISH probe set).

### FISH colocalization

WT WSN virus infected cells were probed with different combinations of FISH probes (Table S1) to assess colocalization between individual segments. Cells were imaged using the sequential scanning program on the Leica SP5 white light laser confocal microscope. To obtain close to Nyquist sampling, stacks of each cell were taken with 0.17 µm separation between slices and the height of the stacks was determined for each cell based on the FISH staining. Images were acquired at 1024×1024 pixel resolution with a 63× Leica objective with a numerical aperture (NA) of 1.4 and a zoom of 4; the resulting pixel size was ∼50×50×168 nm. Background subtraction and subsequent deconvolution of each stack was performed manually for each channel using Huygens Essential software (Version 4.3.1p3, Scientific Volume Imaging BV, Hilversum, Netherlands).

Imaris software (version 7.6.3, Bitplane AG, Zurich, Switzerland) was used for 3D reconstruction, particle tracking, colocalization and statistical analysis. Pearson coefficients in colocalized volumes were obtained between each vRNA probe set from the deconvolved images using Imaris colocalization analysis. The DAPI stain was used to create a channel mask (smoothing = 0.9) to exclude the vRNA nuclear stain from the colocalization quantification. Thus, we were able to compare the staining of different vRNA segments in the cytoplasm of an infected cell. At least 3 different cell volumes were analyzed for each vRNA pair.

To determine the vRNA composition and distance from the nucleus of each cytoplasmic focus we used the Imaris software to create ‘Spots’ from the signal of each channel in the cytoplasm (using the nuclear mask created earlier). All spots from each channel were merged into one ‘Spots’ channel, which created a single spot for every cytoplasmic focus within an infected cell, with intensity values for each channel. This information was used to determine the number of spots positive for all 4 channels versus 3 channels, 2 channels, or a single channel. The frequency of spots from three independent cells was recorded; the numbers were compared using column statistical comparison and a Chi-Squared test using the graphing software, Prism (version 5.0, GraphPad, San Diego, CA), to determine if there was a significant difference between any of the distributions. A separate surface to define the boundary of the cell was created manually based on the vRNA staining pattern in every z-plane. The distance from the nucleus, defined by DAPI staining, was calculated for each spot within this surface and the frequency of spots in 100 nm distance increments was recorded from four independent cells.

### FISH probe specificity

To confirm the specificity of each FISH probe set, two coverslips of A549 cells were transfected with a pPol1 plasmid expressing each vRNA segment of the WSN virus using Fugene HD (Promega, Madison, WI). Cells were fixed 24 hours post-transfection and processed for FISH staining as described above. Transfected cells were stained with FISH probe reactions B and E (Table S1) to ensure coverage of all 8 vRNA segments. FISH stained slides were visualized on a Leica SP5 white light laser microscope using the sequential parameters described above for four color FISH.

### Visualization of Rab11a

Plasmids expressing Rab11a GFP were a kind gift from Dr. Julie Donaldson, National Heart Lung and Blood Institute (NHLBI) and were transfected into A549 cells using Fugene HD transfection reagent (Promega, Madison, WI). 24 hours post-transfection the cells were infected with WT WSN virus (MOI = 3) for subsequent FISH analysis as described above. Images of cells transfected with Rab11a-GFP and stained for vRNA segments were taken on a Leica SP5 confocal microscope using a 63× Leica objective with NA of 1.4.

### Live cell imaging

#### iSPIM

MDCK or A549 cells were seeded onto rectangular coverslips (VWR, 48393-241) and then placed into an imaging chamber (Applied Scientific Instrumentation, I-3078-2450) that fit into a custom-built inverted selective plane illumination microscope (iSPIM). Cells were infected in the chamber at an MOI = 5 and then placed in a 37°C incubator for 16 hours prior to imaging. The iSPIM system was modified from our original design [38], equipped with a 0.3 NA excitation objective (Nikon 10×, 3.5 mm working distance) and a higher, 1.0 NA detection objective (Zeiss, W Plan-Apochromat 63×, 2.1 mm working distance) to increase spatial resolution and fluorescence signal collection. The 488 nm excitation power was set at 50 µW (as measured before the excitation objective), and an electron-multiplying charge coupled device (EM-CCD, Andor, iXon DU-897T) was used to capture fluorescence. We recorded 40 planes per volume for each cell, spacing planes every 1 µm. Volumes were acquired every 2 seconds for a total of 30 minutes.

#### Point scanning confocal microscopy

The spread of influenza was captured on a Leica SP5 point scanning confocal microscope using a 1.4 NA 63× objective. MDCK cells were seeded into an 8-well chambered coverglass slide (Nunc, Rochester, NY) and infected with an MOI = 0.1 with WSN PA-GFP virus. Four hours post-infection, the slides were placed onto the microscope inside a temperature (37°C) and 5% CO_2_ controlled incubator (Microscope incubator S-2, PeCon GmbH, Erbach, Germany). We captured 5 slices per field of view, with ∼1 µm separation between slices, every 10 min for 16 hours. The DIC channel was used to determine stack height.

### Tracking cytoplasmic PA-GFP

3D image stacks captured using the iSPIM were background subtracted and deconvolved using the Huygens software package (SVI, Huygens Professional) using the ‘Classic MLE’ setting with 40 iterations per deconvolution. The resulting datasets were analyzed in ImageJ, generating maximum intensity projections (as displayed in Figure 6A) and axial intensity profiles (Figure 6D). In order to track the motion of cytoplasmic foci, deconvolved datasets were loaded into Imaris for tracking. Manual tracking was performed using the ‘Spots’ function under the Imaris Surpass toolbox and the location of a PA-GFP focus was determined over all frames. A minimum of 30 tracks were collected for each cell type. Statistics for tracks, including the mean track speed, track duration, intensity of the focus over the length of the track, and the xyz position of each focus in each frame throughout the trace were calculated using Imaris. The trajectories for each focus were next analyzed in MATLAB using custom software in order to calculate the mean squared displacements (MSD) vs. time lag (Figure S4). Fusion and transient colocalization events were tallied. Fusions were classified as events wherein two foci came together in a diffraction limited spot and subsequently moved together as a single particle, and confirmed by assessing the axial intensity profile of the particle (Figure 6D). Transient colocalizations were scored as events where two foci came together within a diffraction limited spot for a limited period of time, and did not move together afterwards.

## Supporting Information

Figure S1 **Minimal bleed-through between four FISH visualization channels.** MDCK cells infected with WT WSN (MOI = 3) for 8 hpi were probed with a single FISH probe and visualized with the four-color imaging parameters. Fluorescence of each probe was only seen in the specified channel, demonstrating our ability to spectrally separate all four FISH probe fluorophores. All scale bars are 10 µm.
(PDF)

Figure S2 **WSN vRNA FISH probe specificity.** A549 cells were transfected with individual WSN vRNA Pol1 expression plasmids. Twenty-four hours post-transfection, cells were probed for all eight vRNA segments using the FISH assay. Only cells transfected with the pPol1 plasmid expressing the vRNA that corresponded to the FISH probe had any detectable fluorescence staining. The distribution of vRNA in the transfected cells was different from infected cells, likely because a typical RNP structure was not formed due to lack of NP and polymerase expression. All scale bars are 5 µm.
(PDF)

Figure S3 **The majority of cytoplasmic foci contain at least 4 vRNA segments.** The number of total foci containing 1, 2, 3 or 4 vRNA segments were quantified for MDCK cells (MOI = 3) for 8 hpi stained with probe reactions A, C, D, E and F listed on Table S1. Note that Figure 2B depicts the composition of cells stained with probe B. Each bar represents the percent of foci that contained either 1, 2, 3 or all 4 labeled vRNA segments and is an average of three independent cells that each contained between 1,000–4,000 distinct cytoplasmic foci. The standard error is indicated on each bar.
(PDF)

Figure S4 **Cytoplasmic localization of WSN PA-GFP, NP, and vRNA is CRM1 dependent.** Visualization of PA-GFP, PB2 vRNA segment, and HA vRNA segment in MDCK cells infected with WSN PA-GFP virus and treated with or without leptomycin B (LMB) (A). All scale bars are 5 µm. The percent of WSN PA-GFP infected MDCK cells with cytoplasmic staining of PA-GFP, PB2 vRNA segment, or α-NP in the presence or absence of LMB was calculated (B). Percentage is based on at least 40 cells.
(PDF)

Figure S5 **Mean squared displacement (MSD) curves for PA-GFP tracks in MDCK and A549 cells.** The MSD over time was calculated for each track from MDCK and A549 cells and a representative track demonstrating active transport (A and C) and diffusive transport (B and D) are presented. Polynomial or linear lines of best-fit, dashed black line on each graph, are shown on active or diffusive curves respectively. The equation for the line of best-fit and R-value are displayed and was used to confirm whether the trajectory was active or diffusive. The standard deviation is presented for each time lag.
(PDF)

Figure S6 **Colocalization of influenza vRNA with Rab11a.** A549 cells were transfected with Rab11a-GFP and then infected with WT WSN (MOI = 1). Cells were probed 16 hpi for PB2 vRNA segment using FISH. The images on the right are enlarged from the area denoted by the dashed box. All scale bars are 10 µm.
(PDF)

Movie S1 **iSPIM movie of MDCK cells infected with WSN PA-GFP.** MDCK cells were infected for 16 hours and imaged for 30 min with an entire cell volume captured every 2 seconds. Scale bar: 10 µm.
(AVI)

Movie S2 **iSPIM movie of A549 cells infected with WSN PA-GFP.** A549 cells were infected for 16 hours and then imaged for 30 min with an entire cell volume captured every 2 seconds. Scale bar: 10 µm.
(AVI)

Movie S3 **Overnight confocal movie of WSN PA-GFP spread in MDCK cells.** MDCK cells were infected with WSN PA-GFP (MOI = 0.1) and imaging was initiated 4 hpi. A z stack (5 slices) was taken every 10 min for 16 hours with the cells maintained in a temperature and CO_2_ controlled microscope chamber. The GFP and DIC channels are overlaid to allow for identification of infected cells. This movie displays the spread of WSN PA-GFP and the initiation of infection. Scale bar: 5 µm.
(AVI)

Table S1 **Strategy for multiplexing FISH probes to compare all vRNA segments to each other.**
(PDF)

Table S2 **Number of transient colocalization and fusion events in PA-GFP tracks in MDCK cells.**
(PDF)

Table S3 **Number of transient colocalization and fusion events in PA-GFP tracks in A549 cells.**
(PDF)
