# Correction: Influenza A Virus Assembly Intermediates Fuse in the Cytoplasm

**DOI:** 10.1371/journal.ppat.1006121

**Published:** 2016-12-27

**Authors:** Seema S. Lakdawala, Yicong Wu, Peter Wawrzusin, Juraj Kabat, Andrew J. Broadbent, Elaine W. Lamirande, Ervin Fodor, Nihal Altan-Bonnet, Hari Shroff, Kanta Subbarao

Through continued research, the authors were made aware of a feature in the software used to generate some data and figures that requires a correction. The data analysis regarding the composition of the cytoplasmic foci that alter the total number of foci containing either 1, 2 3 or 4 viral RNA (vRNA) segments stained using four-color FISH requires revision. The vRNA composition of cytoplasmic foci was determined by generating spots for each labeled vRNA channel in Imaris software and then merging all 4 spot channels into a single ‘merged spots’ channel. The authors were not aware that the ‘merge spots’ feature in Imaris is an additive function and thus the merged spot channel double counted all spots with two vRNA segments, triple counted spots with 3 vRNA segments, and quadruple counted spots with 4 vRNA segments. To correct for the over counting, the authors have recalculated the data from the original analysis by dividing the number of spots positive for 2, 3, or 4 vRNA segments by 2, 3 or 4 respectively.

After the recalculation, the revised figures for the composition of the total vRNA-containing foci show that ~40% of the cytoplasmic foci contain only one of the labeled vRNA segments. This solidifies the author’s conclusion that the vRNA segments are not traveling from the nucleus to the plasma membrane as a complex of all 8 segments. In addition, the revised analysis on the vRNA composition of foci as a distance from the nucleus demonstrates that 20% of the foci at the nuclear periphery (0-100nm) contain only one of the labeled vRNA segments. Therefore, the majority (80%) of the foci contain more than one labeled vRNA segment, suggesting that the segments are not exported from the nucleus individually. The reanalysis does not affect the conclusions of the paper. The corrected Fig 2 and S3 Fig are provided here.

**Fig 2:**
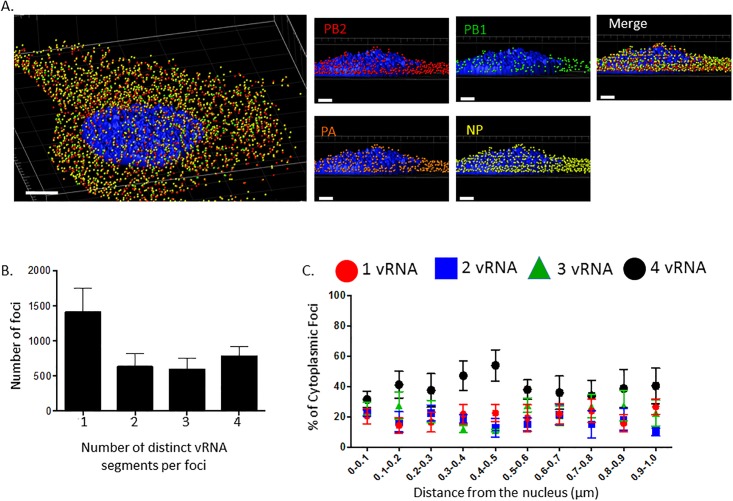
Viral RNA composition and spatial location of cytoplasmic foci. 3D rendering of an MDCK cell infected with WT WSN (MOI = 3) for 8 hpi were stained with probe B from Table S1: WSN PB2 Quasar 570 (red spots), PB1 Alexa-Fluor 488 (green spots), PA Cal Fluor Red 590 (orange spots), and NP Quasar 670 (yellow spots) (A). The DAPI-stained nucleus is labeled in blue. An enlarged region of the 3D image was tilted 90° in the z-direction to provide an axial-view of the cytoplasmic foci and nucleus. Scale bars are 4 μm in the whole cell image and 2 μm in the rotated images. The total number of foci containing 1, 2, 3, or 4 vRNA segments was quantified within a given cell (B). Each bar represents the average from 3 independently analyzed cells with standard error indicated. The distance from the nucleus for each focus from four independent cells was calculated (C). The proportion of foci containing 4,3,2,or 1 distinct vRNA segment with a given range from the nucleus is represented graphically as a scatter plot. Each spot is an average from 4 independent cells and the standard error is indicated.

## Supporting Information

S3 Fig Composition of cytoplasmic foci in cells stained with multiplexed four vRNA segments. The number of total foci containing 1, 2, 3 or 4 vRNA segments were quantified for MDCK cells (MOI = 3) for 8hpi stained with probe reactions A, C, D, E and F listed on Table S1. Note that Fig 2B depicts the composition of cells stained with probe B. Each bar represents the percent of foci that contained either 1, 2, 3 or all 4 labeled vRNA segments and is an average of three independent cells. The standard error is indicated on each bar.
(TIF)
