# Supplementary figures and images for: Influenza A Virus Assembly Intermediates Fuse in the Cytoplasm
Source: PLoS Pathog. 2014 Mar 6;10(3):e1003971. doi: 10.1371/journal.ppat.1003971 (PMC3946384; doi:10.1371/journal.ppat.1003971)

Figure S1: Minimal bleed-through between four FISH visualization channels

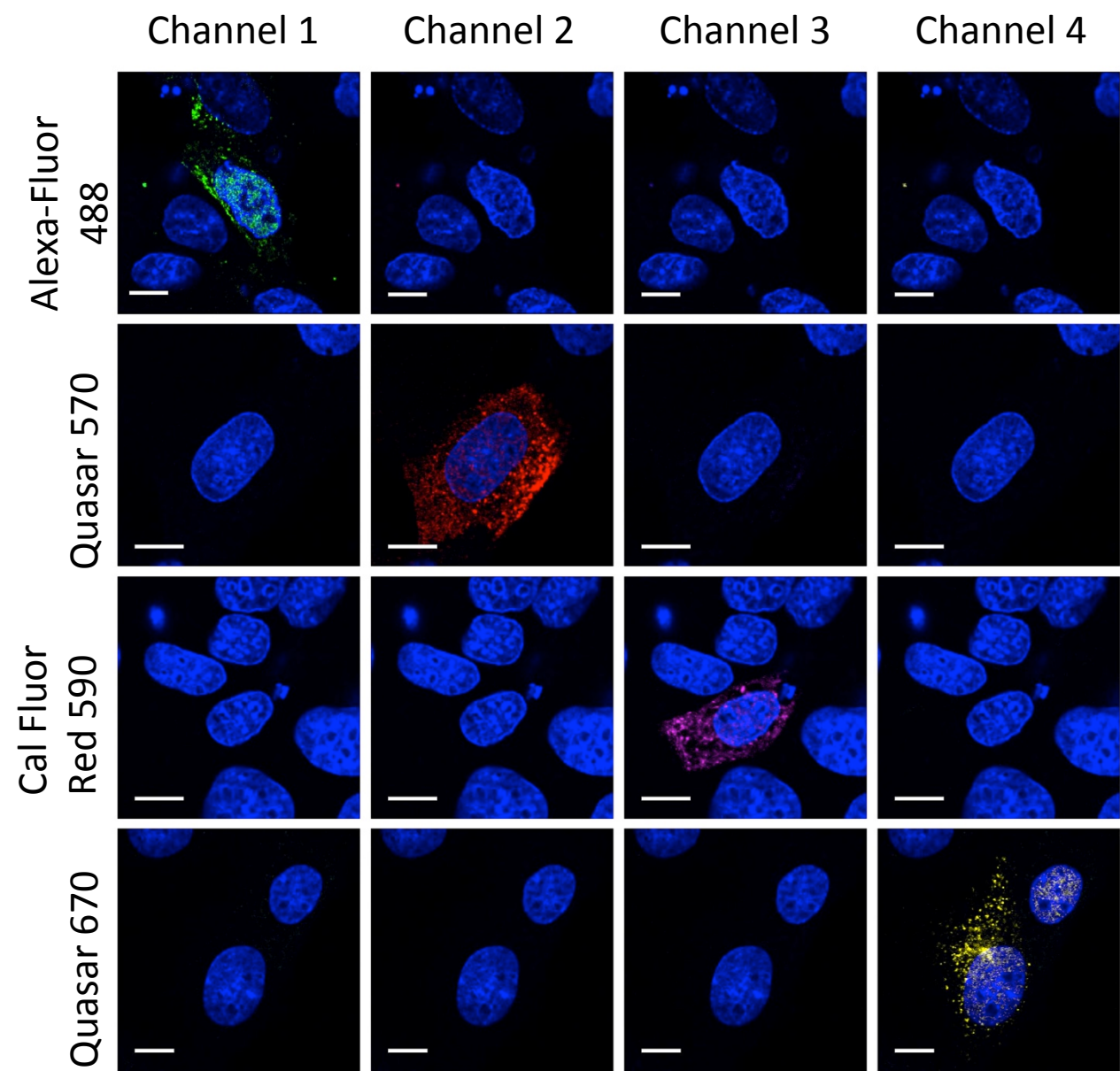

Supplement: Figure S1 — Minimal bleed-through between four FISH visualization channels. MDCK cells infected with WT WSN (MOI = 3) for 8 hpi were probed with a single FISH probe and visualized with the four-color imaging parameters. Fluorescence of each probe was only seen in the specified channel, demonstrating our ability to spectrally separate all four FISH probe fluorophores. All scale bars are 10 µm. (PDF) [file ppat.1003971.s001.pdf]

Figure S2: WSN vRNA FISH probe specificity

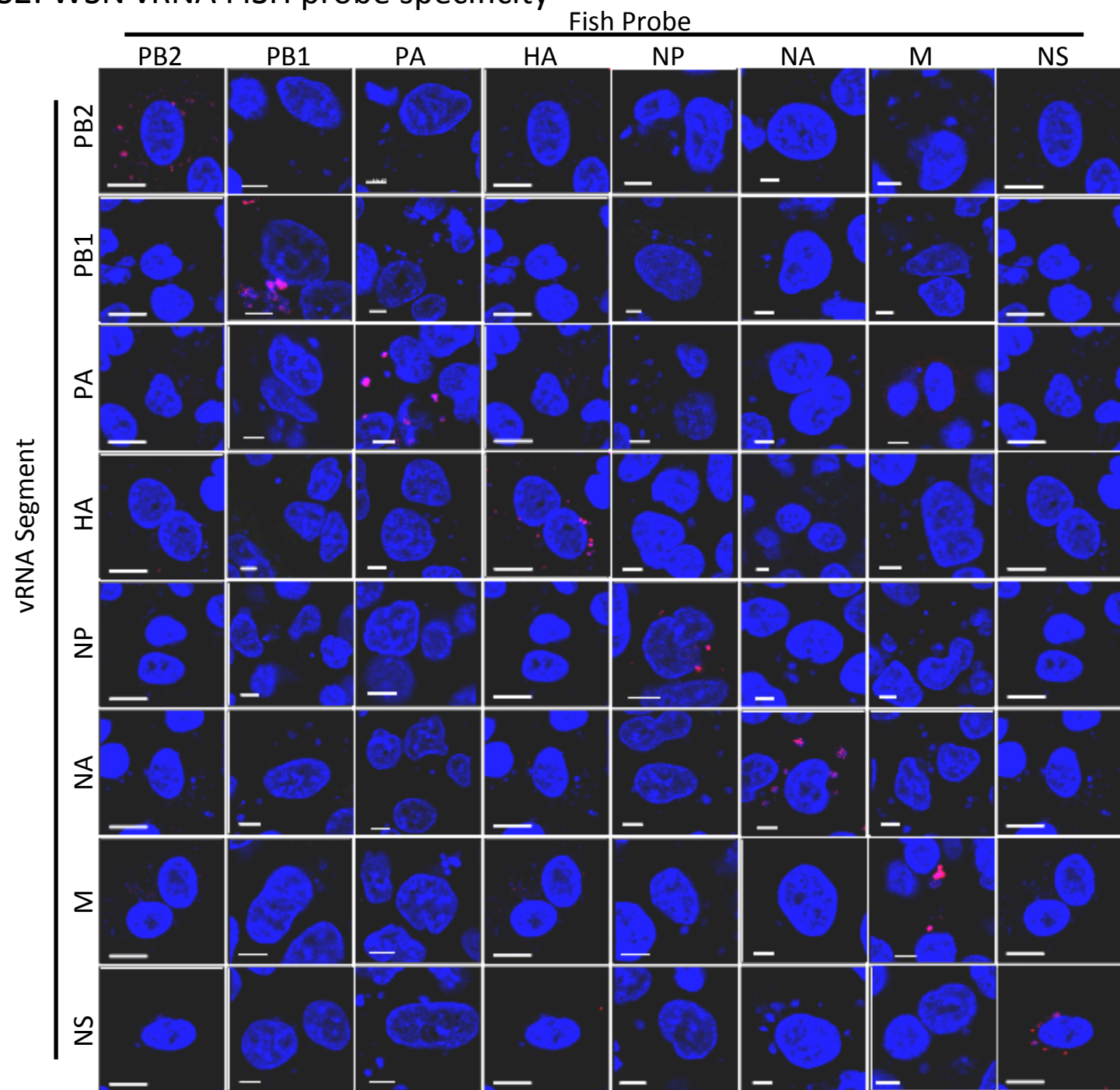

Supplement: Figure S2 — WSN vRNA FISH probe specificity. A549 cells were transfected with individual WSN vRNA Pol1 expression plasmids. Twenty-four hours post-transfection, cells were probed for all eight vRNA segments using the FISH assay. Only cells transfected with the pPol1 plasmid expressing the vRNA that corresponded to the FISH probe had any detectable fluorescence staining. The distribution of vRNA in the transfected cells was different from infected cells, likely because a typical RNP structure was not formed due to lack of NP and polymerase expression. All scale bars are 5 µm. (PDF) [file ppat.1003971.s002.pdf]

Figure S3: The majority of cytoplasmic foci contain at least four distinct vRNA segments

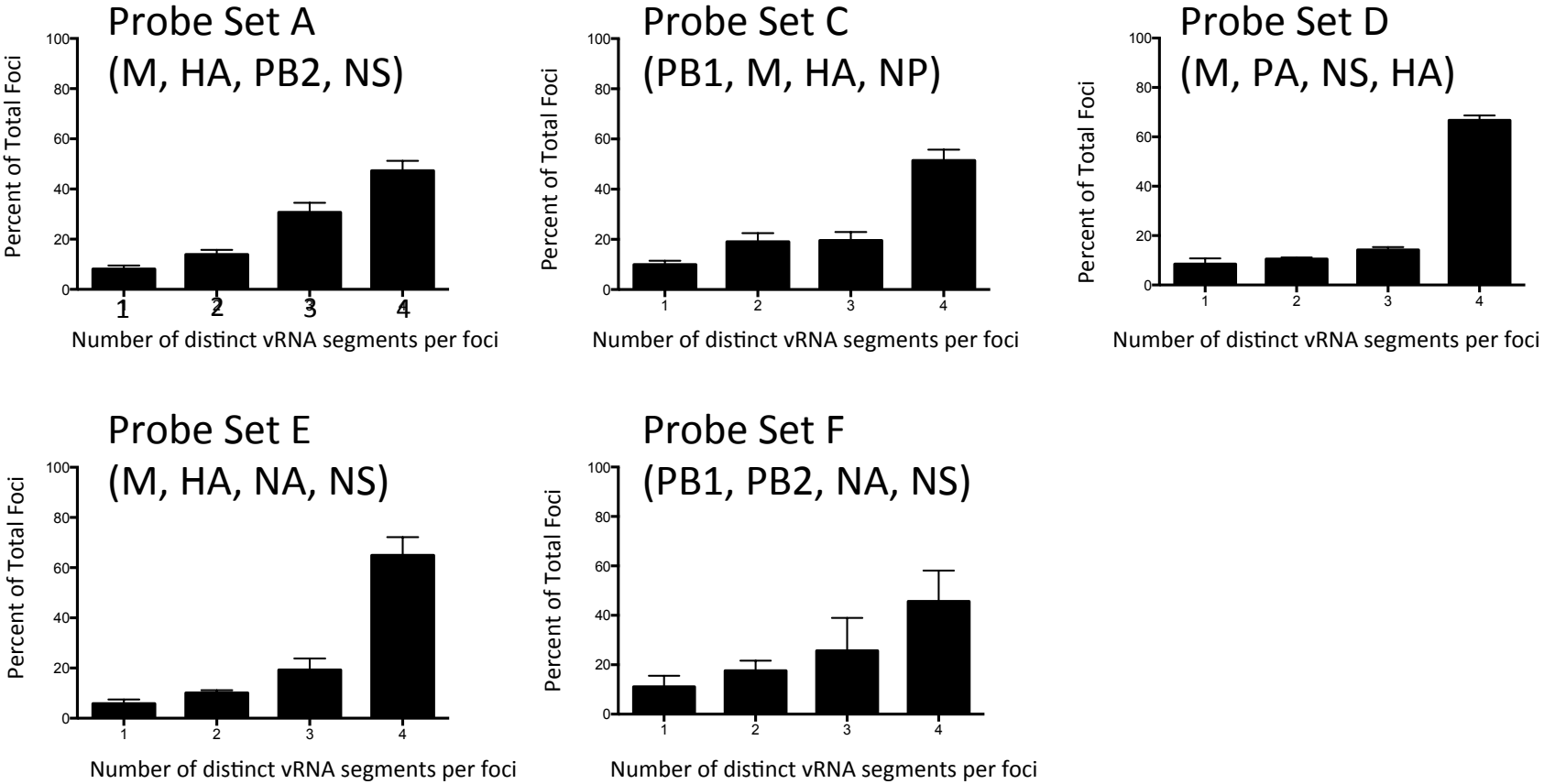

Supplement: Figure S3 — The majority of cytoplasmic foci contain at least 4 vRNA segments. The number of total foci containing 1, 2, 3 or 4 vRNA segments were quantified for MDCK cells (MOI = 3) for 8 hpi stained with probe reactions A, C, D, E and F listed on Table S1. Note that Figure 2B depicts the composition of cells stained with probe B. Each bar represents the percent of foci that contained either 1, 2, 3 or all 4 labeled vRNA segments and is an average of three independent cells that each contained between 1,000–4,000 distinct cytoplasmic foci. The standard error is indicated on each bar. (PDF) [file ppat.1003971.s003.pdf]

Figure S5: Mean squared displacement (MSD) curves for PA-GFP foci in MDCK and A549 cells

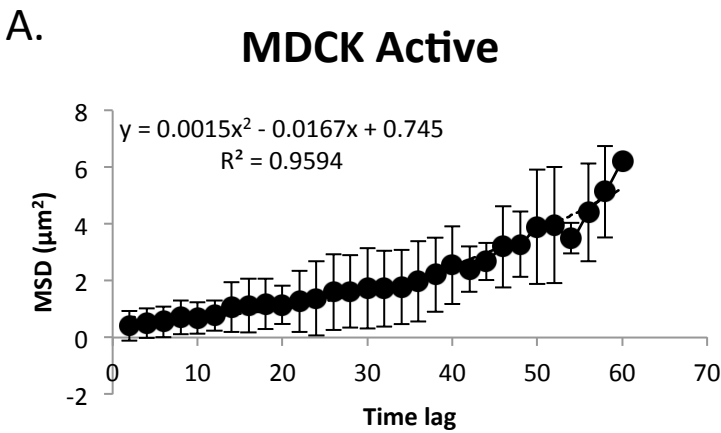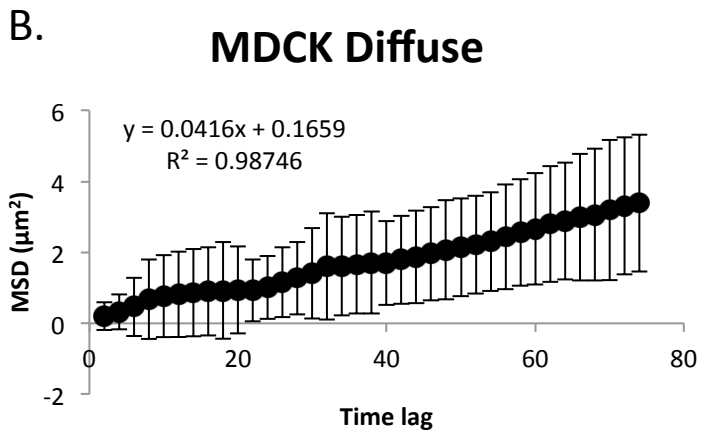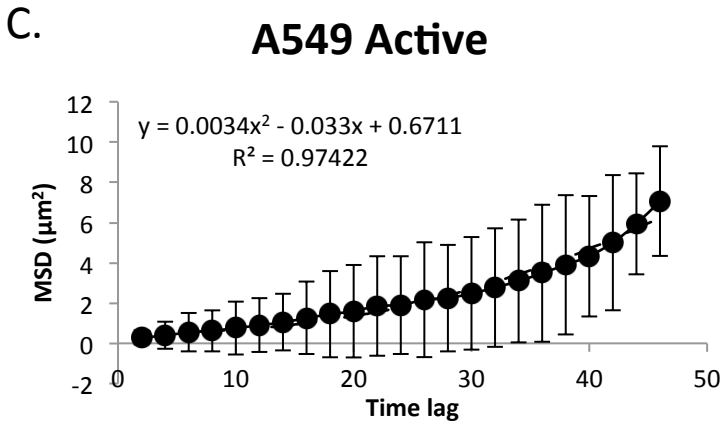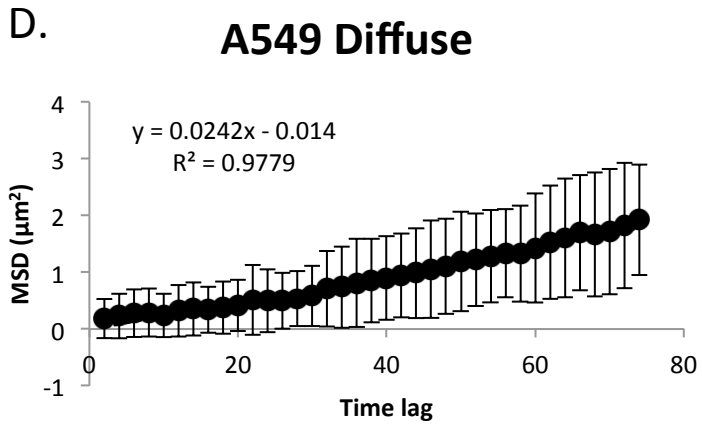

Supplement: Figure S5 — Mean squared displacement (MSD) curves for PA-GFP tracks in MDCK and A549 cells. The MSD over time was calculated for each track from MDCK and A549 cells and a representative track demonstrating active transport (A and C) and diffusive transport (B and D) are presented. Polynomial or linear lines of best-fit, dashed black line on each graph, are shown on active or diffusive curves respectively. The equation for the line of best-fit and R-value are displayed and was used to confirm whether the trajectory was active or diffusive. The standard deviation is presented for each time lag. (PDF) [file ppat.1003971.s005.pdf]

Figure S6: Colocalization of influenza vRNA with Rab11a

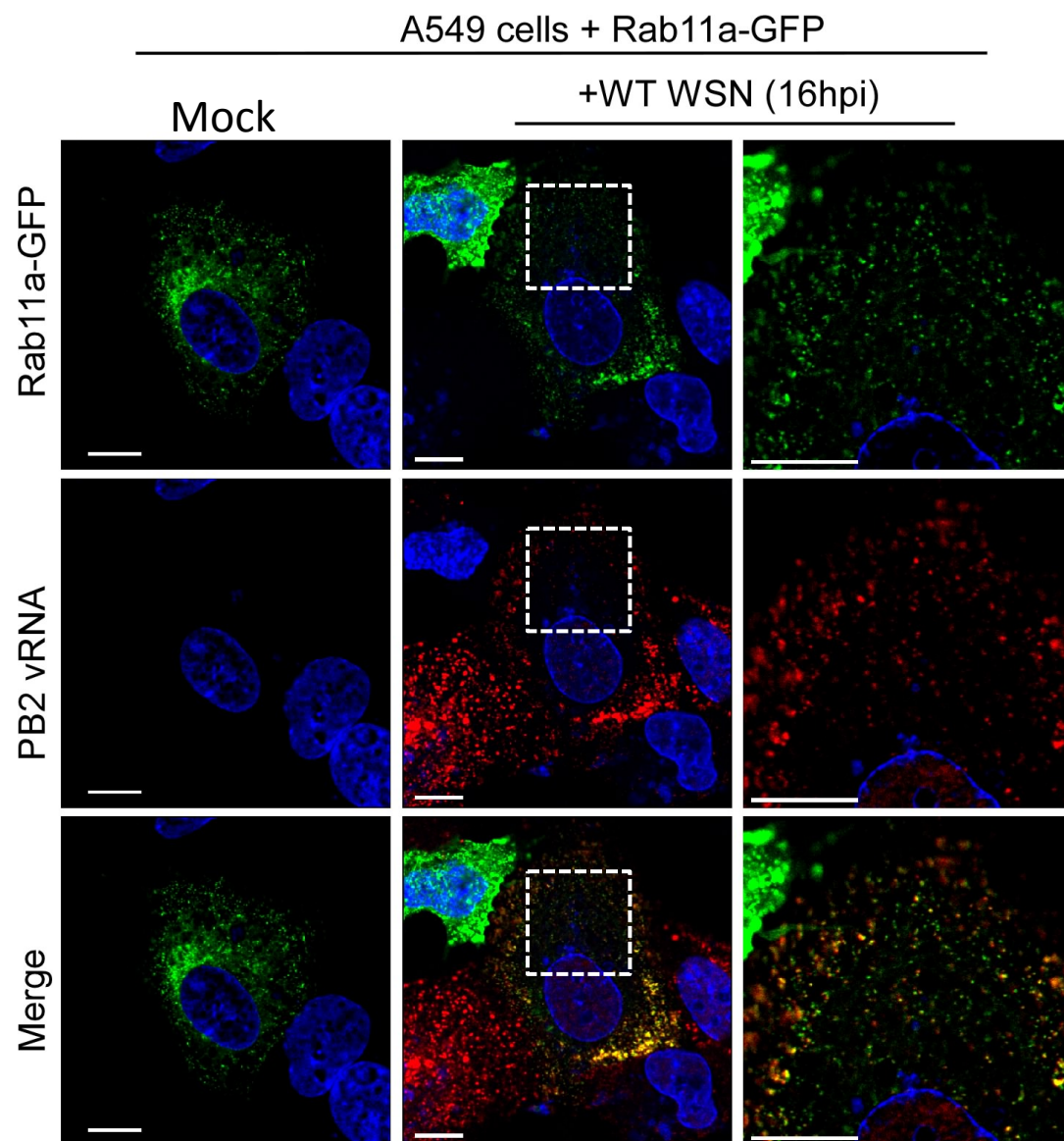

Supplement: Figure S6 — Colocalization of influenza vRNA with Rab11a. A549 cells were transfected with Rab11a-GFP and then infected with WT WSN (MOI = 1). Cells were probed 16 hpi for PB2 vRNA segment using FISH. The images on the right are enlarged from the area denoted by the dashed box. All scale bars are 10 µm. (PDF) [file ppat.1003971.s006.pdf]
