# Supplementary material for: Influenza A Virus Assembly Intermediates Fuse in the Cytoplasm
Source: PLoS Pathog. 2014 Mar 6;10(3):e1003971. doi: 10.1371/journal.ppat.1003971 (PMC3946384; doi:10.1371/journal.ppat.1003971)

Figure S4: Cytoplasmic localization of WSN PA-GFP, NP, and vRNA is CRM1 dependent

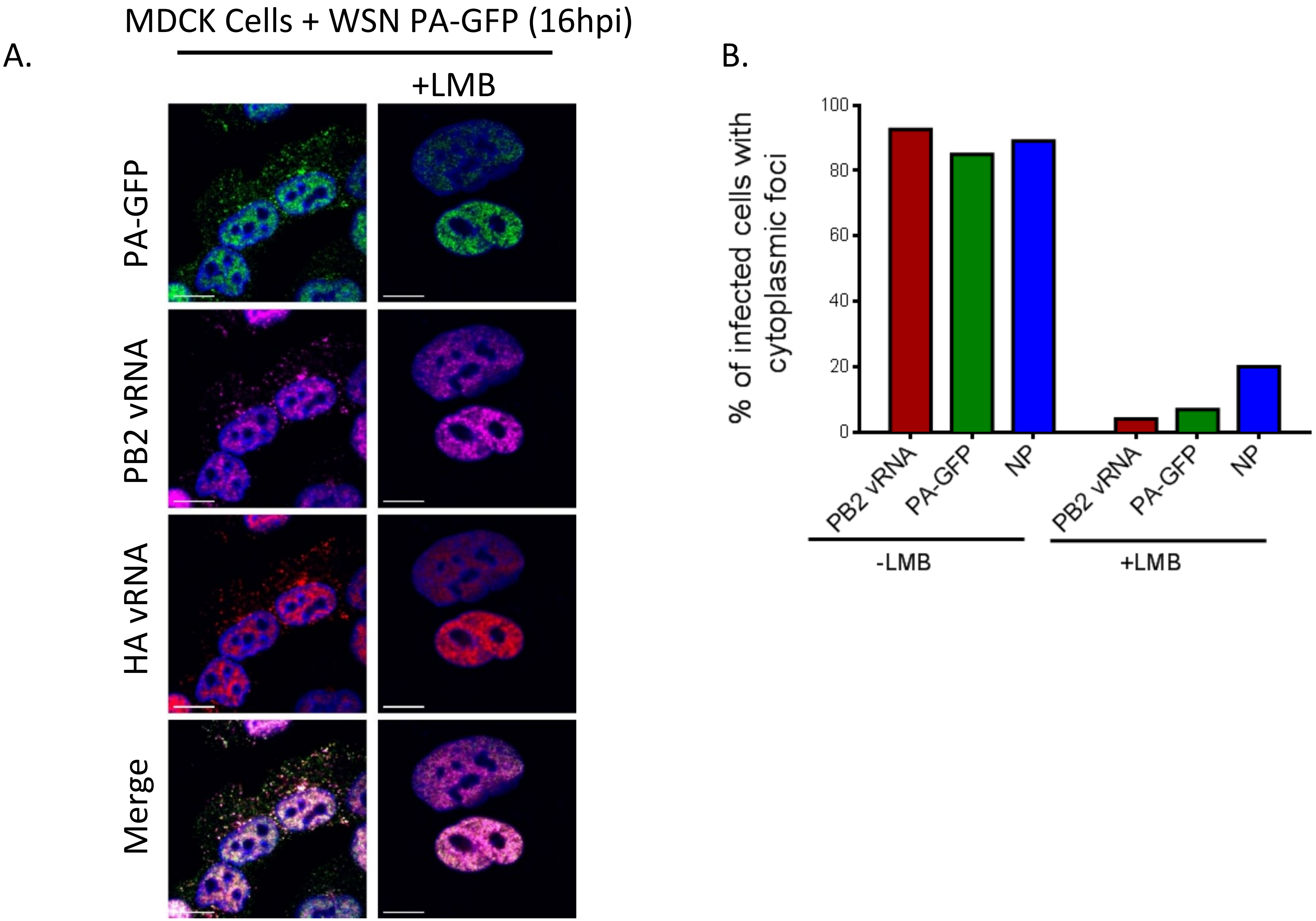

Supplement: Figure S4 — Cytoplasmic localization of WSN PA-GFP, NP, and vRNA is CRM1 dependent. Visualization of PA-GFP, PB2 vRNA segment, and HA vRNA segment in MDCK cells infected with WSN PA-GFP virus and treated with or without leptomycin B (LMB) (A). All scale bars are 5 µm. The percent of WSN PA-GFP infected MDCK cells with cytoplasmic staining of PA-GFP, PB2 vRNA segment, or α-NP in the presence or absence of LMB was calculated (B). Percentage is based on at least 40 cells. (PDF) [file ppat.1003971.s004.pdf]
