# Supplementary material for: Influenza A Virus Assembly Intermediates Fuse in the Cytoplasm
Source: PLoS Pathog. 2014 Mar 6;10(3):e1003971. doi: 10.1371/journal.ppat.1003971 (PMC3946384; doi:10.1371/journal.ppat.1003971)
Supplement: Table S1 — Strategy for multiplexing FISH probes to compare all vRNA segments to each other. (PDF) [file ppat.1003971.s010.pdf]

Table S1: Strategy for multiplexing FISH probes to compare all vRNA segments to each other

Multi Color Reactions	Channels			
	1	2	3	4
A	M	HA	PB2	NS
B	PB1	PB2	PA	NP
C	PB1	M	HA	NP
D	M	PA	HA	NS
E	M	HA	NA	NS
F	PB1	PB2	NA	NS
G		PA	NA	NP
H			NP	NS
