# Supplementary material for: Influenza A Virus Assembly Intermediates Fuse in the Cytoplasm
Source: PLoS Pathog. 2014 Mar 6;10(3):e1003971. doi: 10.1371/journal.ppat.1003971 (PMC3946384; doi:10.1371/journal.ppat.1003971)
Supplement: Table S2 — Number of transient colocalization and fusion events in PA-GFP tracks in MDCK cells. (PDF) [file ppat.1003971.s011.pdf]

Table S2: Number of transient colocalization and fusion events in PA-GFP tracks in MDCK cells

Track Number	Colocalization Events		Track Duration (sec)
	Fusions	Transient Colocalizations	
1	1	1	18.00
2	0	1	42.00
3	0	0	10.00
4	1	2	42.00
5	0	1	14.00
6	0	1	88.00
7	0	0	14.00
8	0	2	26.00
9	0	1	18.00
10	0	1	14.00
11	0	2	62.00
12	0	0	36.00
13	0	0	44.00
14	0	0	80.00
15	0	1	20.00
16	0	1	38.00
17	1	2	80.00
18	0	1	62.00
19	0	2	100.00
20	0	2	40.00
21	0	1	48.00
22	0	0	32.00
23	0	0	38.00
24	0	1	20.00
25	0	0	70.00
26	0	0	42.00
27	0	1	24.00
28	0	0	86.00
29	0	2	72.00
30	0	1	34.00
31	1	1	48.00
32	0	0	94.00
33	0	1	10.00
34	1	1	46.00
35	0	1	44.00
36	0	0	18.00
37	0	1	52.00
38	0	1	40.00
39	0	1	24.00
40	1	2	32.00
41	0	1	24.00
42	0	1	60.00
43	0	0	34.00
44	0	0	10.00
45	0	1	22.00
