# Supplementary material for: Influenza A Virus Assembly Intermediates Fuse in the Cytoplasm
Source: PLoS Pathog. 2014 Mar 6;10(3):e1003971. doi: 10.1371/journal.ppat.1003971 (PMC3946384; doi:10.1371/journal.ppat.1003971)
Supplement: Table S3 — Number of transient colocalization and fusion events in PA-GFP tracks in A549 cells. (PDF) [file ppat.1003971.s012.pdf]

Table S3: Number of transient colocalization and fusion events in PA-GFP tracks in A549 cells

Track Number	Colocalization Events		Track Duration (sec)
	Fusions	Transient Colocalizations	
1	0	2	45.00
2	0	1	32.00
3	0	1	17.00
4	0	1	44.00
5	0	0	46.00
6	0	1	23.00
7	0	1	27.00
8	0	1	19.00
9	1	2	31.00
10	1	1	25.00
11	0	1	19.00
12	0	1	20.00
13	0	1	19.00
14	0	1	58.00
15	0	0	18.00
16	0	1	18.00
17	0	1	62.00
18	0	1	20.00
19	0	1	8.00
20	2	2	126.00
21	0	1	128.00
22	0	1	64.00
23	0	1	34.00
24	0	0	48.00
25	0	1	88.00
26	0	1	114.00
27	0	1	156.00
28	0	1	116.00
29	0	1	34.00
30	0	1	50.00
31	0	0	136.00
32	0	1	80.00
33	0	2	74.00
