# Supplementary figures and images for: Correction: Influenza A Virus Assembly Intermediates Fuse in the Cytoplasm
Source: PLoS Pathog. 2016 Dec 27;12(12):e1006121. doi: 10.1371/journal.ppat.1006121 (PMC5189951; doi:10.1371/journal.ppat.1006121)

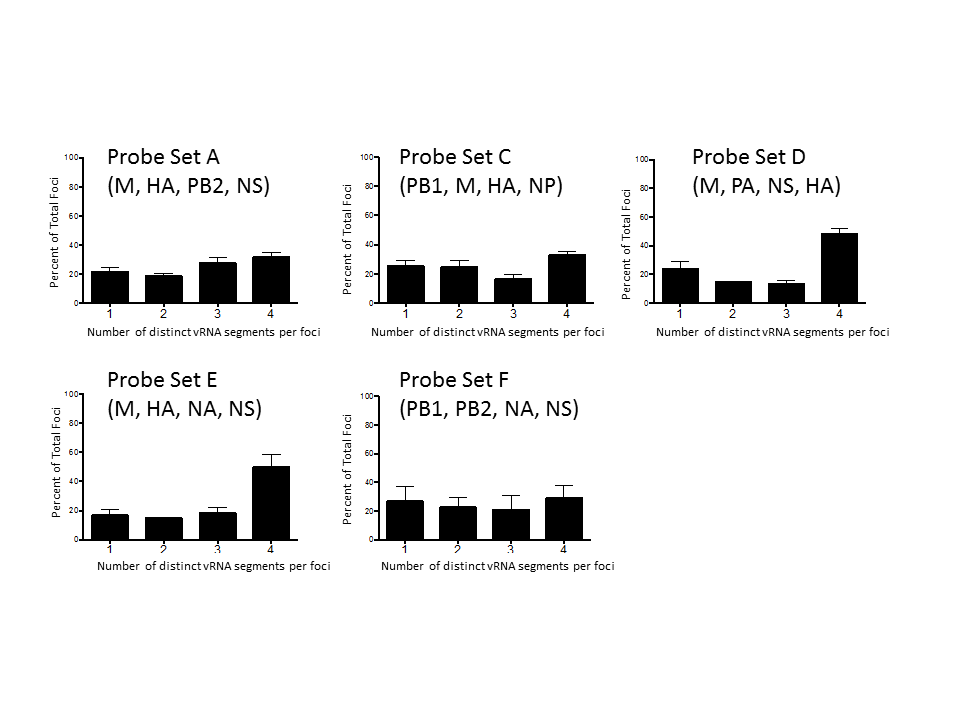

Supplement: S3 Fig — The number of total foci containing 1, 2, 3 or 4 vRNA segments were quantified for MDCK cells (MOI = 3) for 8hpi stained with probe reactions A, C, D, E and F listed on Table S1. Note that Fig 2B depicts the composition of cells stained with probe B. Each bar represents the percent of foci that contained either 1, 2, 3 or all 4 labeled vRNA segments and is an average of three independent cells. The standard error is indicated on each bar. (TIF) [file ppat.1006121.s001.tif]
